# Esketamine Nasal Spray in the Treatment of Depression: A Comprehensive Analysis of its Efficacy and Safety in 107 Patients of Sulwan Psychiatric Hospital in Kingdom of Bahrain

**DOI:** 10.1192/j.eurpsy.2025.1299

**Published:** 2025-08-26

**Authors:** A. M. Al Hamada

**Affiliations:** Psychiatry, Sulwan Psychiatric Hospital, Manama, Bahrain

## Abstract

**Introduction:**

Depression is one of the most disabling diseases worldwide, but despite numerous attempts to investigate novel drugs, no significant step forward has been taken so far. Traditional antidepressants, including selective serotonin reuptake inhibitors and serotonin and norepinephrine reuptake inhibitors, have been shown to have a slow onset of action and require assessing these drugs for up to eight weeks to determine their effectiveness. As a result, there is an urgent medical need for new, rapid, and effective treatments like Esketamine Nasal Spray for the large number of patients suffering from the disease.

**Objectives:**

In our study, treated with esketamine nasal spray in an inpatient setting, we were able to show better functioning and severe depression, and, at the same time, a reduction in suicidal thoughts was documented. Our 107 analyzed patients demonstrated that this drug is safe and suitable for short-term use.

**Methods:**

One hundred and seven (107) participants with major depressive episodes (MDE) were examined at Sulwan Psychiatric Hospital. The study, spanning 12 weeks, comprised two treatment protocols. After they had been educated concerning the two groups, the subjects decided their preferred treatment protocols. During the four-week induction phase, 56 mg - 84 mg of drug treatment twice a week was administered to the subjects. Then, based on whether they showed clinical response during the induction phase, the subjects entered the eight-week maintenance phase with 28 mg-56 mg twice monthly.

**Results:**

In total, after 14 administrations, all depressive scores decreased significantly, indicating an improvement in depressive symptoms. Esketamine had a fast onset of action, so that already half of the patients felt better after the 2nd administration and 75% showed an effect after the 4th administration; not only the reduction of the mean but also that of the median illustrated a quick relief.

**Conclusions:**

Our main results confirm the unique contribution of this study. In summary, the intranasal administration of esketamine is effective in patients with treatment-resistant depression (TRD) due to major depressive disorder (MDD) and bipolar disorder (BD).

**Disclosure of Interest:**

None Declared

